# A matter of scale: apparent niche differentiation of diploid and tetraploid plants may depend on extent and grain of analysis

**DOI:** 10.1111/jbi.12663

**Published:** 2015-12-11

**Authors:** Bernhard Kirchheimer, Christoph C. F. Schinkel, Agnes S. Dellinger, Simone Klatt, Dietmar Moser, Manuela Winkler, Jonathan Lenoir, Marco Caccianiga, Antoine Guisan, Diego Nieto‐Lugilde, Jens‐Christian Svenning, Wilfried Thuiller, Pascal Vittoz, Wolfgang Willner, Niklaus E. Zimmermann, Elvira Hörandl, Stefan Dullinger

**Affiliations:** ^1^ Department of Botany and Biodiversity ResearchUniversity of Vienna1030ViennaAustria; ^2^ Department of Systematics, Biodiversity and Evolution of PlantsGeorg‐August‐University of Göttingen37073GöttingenGermany; ^3^ GLORIA co‐ordinationUniversity of Natural Resources and Life Sciences ViennaCenter for Global Change and SustainabilityViennaAustria; ^4^ Institute for Interdisciplinary Mountain ResearchAustrian Academy of SciencesInnsbruckAustria; ^5^ UR Ecologie et Dynamique des Systèmes Anthropisés (EDYSANFRE 3498 CNRS‐UPJV)Jules Verne University of PicardieF‐80037Amiens Cedex 1France; ^6^ Department of BiosciencesUniversity of Milan20133MilanItaly; ^7^ Department of Ecology and EvolutionUniversity of LausanneSwitzerland; ^8^ Institute of Earth Surface DynamicsUniversity of LausanneLausanneSwitzerland; ^9^ Appalachian LaboratoryUniversity of Maryland Center for Environmental ScienceFrostburgMD21532USA; ^10^ Section for Ecoinformatics & BiodiversityDepartment of BioscienceAarhus UniversityDK‐8000Aarhus CDenmark; ^11^ Laboratoire d'Écologie Alpine (LECA)Université Grenoble AlpesF‐38000GrenobleFrance; ^12^ Laboratoire d'Écologie Alpine (LECA)CNRSGrenobleF‐38000France; ^13^ Institute of Earth Surface DynamicsUniversity of LausanneSwitzerland; ^14^ Vienna Institute for Nature Conservation and Analyses1090ViennaAustria; ^15^ Landscape DynamicsSwiss Federal Research Institute WSLBirmensdorfCH‐8903Switzerland; ^16^ Department of Environmental Systems ScienceSwiss Federal Institute of Technology ETHCH‐8092ZürichSwitzerland

**Keywords:** apomixis, alpine plants, competition, European Alps, indicator values, niche breadth, niche shift, polyploidization, *Ranunculus kuepferi*, spatial grain

## Abstract

**Aim:**

Emerging polyploids may depend on environmental niche shifts for successful establishment. Using the alpine plant *Ranunculus kuepferi* as a model system, we explore the niche shift hypothesis at different spatial resolutions and in contrasting parts of the species range.

**Location:**

European Alps.

**Methods:**

We sampled 12 individuals from each of 102 populations of *R. kuepferi* across the Alps, determined their ploidy levels, derived coarse‐grain (100 × 100 m) environmental descriptors for all sampling sites by downscaling WorldClim maps, and calculated fine‐scale environmental descriptors (2 × 2 m) from indicator values of the vegetation accompanying the sampled individuals. Both coarse and fine‐scale variables were further computed for 8239 vegetation plots from across the Alps. Subsequently, we compared niche optima and breadths of diploid and tetraploid cytotypes by combining principal components analysis and kernel smoothing procedures. Comparisons were done separately for coarse and fine‐grain data sets and for sympatric, allopatric and the total set of populations.

**Results:**

All comparisons indicate that the niches of the two cytotypes differ in optima and/or breadths, but results vary in important details. The whole‐range analysis suggests differentiation along the temperature gradient to be most important. However, sympatric comparisons indicate that this climatic shift was not a direct response to competition with diploid ancestors. Moreover, fine‐grained analyses demonstrate niche contraction of tetraploids, especially in the sympatric range, that goes undetected with coarse‐grained data.

**Main conclusions:**

Although the niche optima of the two cytotypes differ, separation along ecological gradients was probably less decisive for polyploid establishment than a shift towards facultative apomixis, a particularly effective strategy to avoid minority cytotype exclusion. In addition, our results suggest that coarse‐grained analyses overestimate niche breadths of widely distributed taxa. Niche comparison analyses should hence be conducted at environmental data resolutions appropriate for the organism and question under study.

## Introduction

Polyploidization is an important mechanism of adaptation and speciation in plants (Levin, 1983; De Bodt *et al*., 2005). Polyploidization often has negative consequences (cf. Comai, 2005), but there are also potential benefits. One important advantage of genome duplication is the possibility of evolving duplicated gene copies to fulfil new or slightly varied functions (neofunctionalization or subfunctionalization) of genes. Such processes may allow for ecological niche expansion or increased flexibility in the organism's response to environmental change (Adams & Wendel, 2005; Lynch, 2007). Moreover, asexual reproduction and the breakdown of self‐incompatibility, which often accompany polyploidization (Barringer, 2007), enable polyploids to reproduce independently of pollinators and mating partners.

As polyploids originate within diploid populations, the establishment of polyploid lineages requires that polyploids avoid minority cytotype exclusion (Levin, 1975) and withstand competition with diploid cytotypes, which are initially present in (much) larger densities (Baack, 2005). A way to avoid competitive exclusion by diploid ancestors is habitat segregation by niche differentiation, which has been invoked as a primary mechanism facilitating polyploid establishment (Husband & Schemske, 2000; Levin, 2004). A number of observations apparently corroborate this ‘niche shift hypothesis’. In particular, polyploids were often reported to occupy physically harsh environments at or beyond the ecological tolerance of their diploid ancestors (e.g. Hagerup, 1932; Kearney, 2005). Nevertheless, experimental tests of niche differentiation among diploids and polyploids, which have mostly been done at much smaller spatial scales, have delivered equivocal results (Baack & Stanton, 2005; Buggs & Pannell, 2007; Raabova *et al*., 2008).

The study of niche differentiation has recently made important progress triggered by the large‐scale availability of climate data and the development of new statistical methods (Wiens & Graham, 2005; Warren *et al*., 2008; Broennimann *et al*., 2012; Petitpierre *et al*., 2012). These data and methods allow for improved measurement of climatic niche overlap between taxa based on information about their distribution in geographical space (Guisan *et al*., 2014). Based on such analysis, the idea of niche shifts as a generic prerequisite of polyploid establishment has recently been challenged (Glennon *et al*., 2014). However, assessing niche differentiation of diploids and polyploids with this approach may require data beyond the most readily available and hence commonly used climatic variables, particularly those provided by WorldClim (Hijmans *et al*., 2005). First, plant taxa may not only differ with respect to their climatic requirements but also with respect to non‐climatic niche dimensions like soil properties, biotic interactions or disturbance frequency. These differences might be particularly important when ranges of taxa are overlapping, and hence climates are similar, such as is the case with many pairs of diploids and polyploids. And second, the commonly used climate data are spatially interpolated from point measures, their resolution is rather coarse, and their accuracy partly questionable (Bedia *et al*., 2013). The climatic conditions indicated by these variables might hence not necessarily be representative of the microenvironment plants experience, particularly in landscapes with pronounced relief (Scherrer & Korner, 2010; Lenoir *et al*., 2013; Glennon *et al*., 2014).

Representing climatic conditions at an appropriate scale and incorporating non‐climatic niche dimensions are hence next steps to improve analyses of niche differentiation between diploid and polyploid plants. Taking these steps is usually limited by data availability but the use of indicator values (e.g. Ellenberg *et al*., 1991; Landolt *et al*., 2010) derived from the vegetation accompanying the species or cytotypes (Felber‐Girard *et al*., 1996; Lenoir *et al*., 2013) offers a way to circumvent this problem. Studying the niches of taxa based on such indicator values has been facilitated with the growing availability of vegetation plot databases which provide fine‐grain vegetation data over a large spatial extent (e.g. Lenoir *et al*., 2012). From these databases, presence–absence information for a species of interest can be derived and indicator values of e.g. temperature, nutrient and water availability or soil acidity (e.g. Ellenberg *et al*., 1991) can be calculated from the accompanying vegetation. In combination, these data allow the comparison of climatic and non‐climatic niches over entire species ranges at fine spatial resolutions. However, comparing the niches of polyploids and their diploid ancestors with this approach requires that vegetation plot data contain sufficient information to distinguish cytotypes within one species – which is rarely the case.

In this study, we focus on one particular model system to develop an in‐depth study of niche differentiation between diploid and tetraploid cytotypes. We try to overcome the aforementioned problems through: (1) consistent sampling and cytotype determination across (nearly) the whole spatial range of the species complex and (2) combining and comparing an analysis based on GIS‐derived coarse‐grained climate data with an analysis based on fine‐grained climatic and non‐climatic indicators derived from a large set of vegetation plots. In addition, we do not only focus on possible differentiation of cytotypes in terms of change in niche optima, but also in terms of change in niche breadth (Boulangeat *et al*., 2012; Theodoridis *et al*., 2013), niche space unique to tetraploids (niche expansion by tetraploids) and niche space unique to diploids (niche unfilling by tetraploids, cf. Guisan *et al*., 2014). Our model system is *Ranunculus kuepferi* Greuter & Burdet, a high‐mountain buttercup native mainly to the European Alps. To analyse niche relationships in this model system, we ask whether (1) diploids and tetraploids differ in the position of their niche optima and/or in their niche breadths; (2) whether these differences vary when comparing sympatric and allopatric diploid and tetraploid cytotypes; (3) whether results based on coarse‐grained data are consistent with results based on indicator‐derived fine‐grained environmental data. We hypothesize that, if niche differentiation is important for tetraploid establishment, the niches of the two cytotypes will differ particularly in their sympatric range, while overlap may increase where diploids are absent. We moreover assume that fine‐grained environmental data deliver a more precise definition of the cytotypes' niches and hence a more pronounced differentiation among them.

## Materials and methods

### Model organism

The perennial herb *Ranunculus kuepferi* occurs in various types of alpine grasslands and pastures at elevations between 1300–2800 m. Autopolyploid cytotypes of the species have repeatedly emerged from diploid ancestors at the south‐western fringes of the European Alps (Burnier *et al*., 2009; Cosendai *et al*., 2011, 2013). While tetraploids have colonized a wide range of the Alps, diploids remained restricted to their glacial refugia (Cosendai & Hörandl, 2010). Tetraploids and diploids differ in their reproductive systems: while diploid populations mostly reproduce by outcrossing, tetraploids mainly reproduce by apomixis, although they are also capable of sexual seed production (facultative apomixis, Cosendai & Hörandl, 2010). Some populations in the geographical contact zone are mixed and in a few populations triploid, pentaploid, and hexaploid individuals occur. Some isolated small tetraploid populations of the species were found in the Apennines and on the island of Corsica (Cosendai & Hörandl, 2010).

### Study area

**Figure 1:**
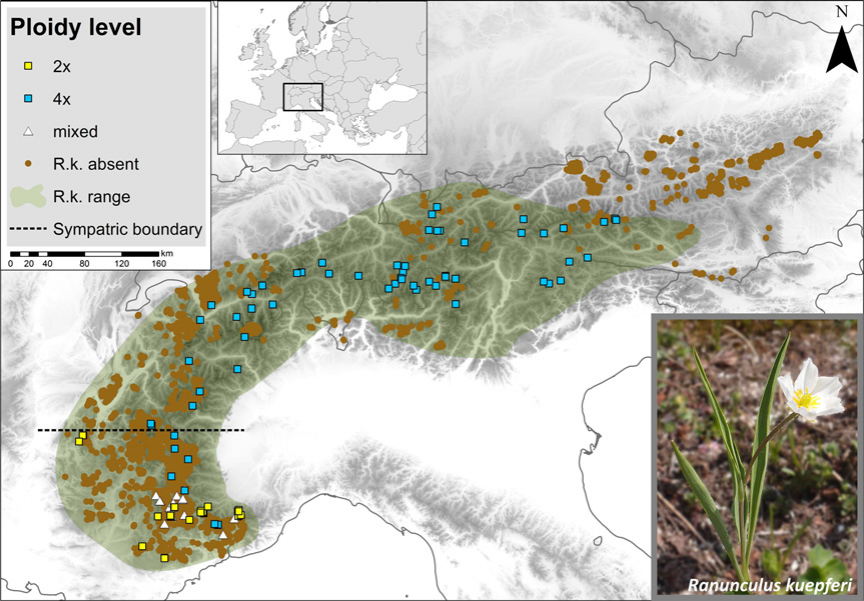
Sampling sites (*N* = 102) in 2013 and 2014. Diploid (2×), tetraploid (4×) and mixed populations are shown in yellow squares, blue squares and white triangles respectively. Vegetation relevés without *Ranunculus kuepferi* taken from the Alps vegetation database are given in brown dots (R.k. absent). Distribution range of *Ranunculus kuepferi* is coloured in green (R.k. range).

The study area constitutes the geographical range of *R. kuepferi* in the European Alps (Fig. 1). Within this area we searched for records of populations in the literature (Cosendai & Hörandl, 2010) as well as in herbaria (see Appendix S1 in Supporting Information). We selected 102 sampling sites from these sources which comprise all known diploid populations and a large set of tetraploids covering the whole Alpine range of the species (Fig. 1).

### Sampling design

During the growing seasons of 2013 and 2014 we searched and sampled all 102 of these populations. Once we had detected the population in the field we chose a plot of 100 × 100 m within which we randomly selected four groups of three *R. kuepferi* individuals. Subsequently, we positioned a frame of 2 × 2 m around each of the four groups, recorded all vascular plants growing within this frame, and collected leaf material from the 12 *R. kuepferi* individuals for determination of ploidy level. Ploidy level was determined by using flow cytometry following methods of Dolezel *et al*. (2007).

### Environmental data

Environmental conditions at the 102 sampling sites were characterized by two different sets of data. The first data set, which we call coarse‐grained environmental data, was derived from WorldClim (Hijmans *et al*., 2005) and included four variables which (1) were not closely correlated (Pearson *r *<* *0.75) and (2) represent important climatic drivers of plant growth (Körner, 2003): temperature [maximum temperature of warmest month (bio5) and annual temperature range (bio7)], water availability [precipitation of driest month (bio14) and an ombrothermic index, Io (see Appendix S1)]. In addition to these climatic variables we added slope inclination (slope) and the percentage area of calcareous substrates (calcium). To generate environmental data resolution matching the size of our sampling plots we statistically downscaled all climatic layers to and computed the soil variables at a resolution of 100 × 100 m. For a detailed description of data preparation see Appendix S1.

The second data set, which we call fine‐grained environmental data, was derived from the species lists of the 2 × 2 m plots and their Landolt indicator values (Landolt *et al*., 2010). Landolt indicator values represent a nine‐level (1, 1.5, 2, … 5.0) ordinal, expert‐based classification of species according to the position of their realized ecological niche along different environmental gradients. Although such indicator values are based on expert judgement and refer to the species' niche optimum only, they have been proven reliable and useful indicators of local‐scale environmental conditions in many studies (e.g. Diekmann, 2003; Lenoir *et al*., 2013). Based on the pooled species lists of the four 2 × 2 m plots we calculated, for each of the 102 populations, a simple unweighted mean of the climatic indicator values for temperature (*T*), the average and the variability in soil moisture during the growing season (F and W respectively), as well as for soil pH (R) and soil nutrient availability (N). *Ranunculus kuepferi* itself was removed from the species lists before calculating indicator values.

### Statistical analysis

For comparing the niches of diploid and tetraploid populations we used an approach introduced by Broennimann *et al*. (2012, 2014: ‘PCA‐env’). The method first translates a multivariable environmental space into a two‐dimensional one by means of a principal components analysis (PCA). PCA space is then gridded and smoothed densities of taxa, in our case cytotype occurrences, as well as smoothed densities of environmental conditions in the study area (=background conditions) are calculated for each grid cell by a kernel density function. Finally, the species' density function is divided by the background area's density function to derive a description of the (realized) species niche which is independent of sampling effort (number of occurrences) and accounts for environmental availability in the study area (Guisan *et al*., 2014). As a corollary, however, the method does not only need environmental information about the sampling sites of species, but also on the availability of environmental conditions in the background area. In addition to our sampling plots, we hence used a set of vegetation relevés from the European Alps to characterize the density of environmental conditions across the Alpine range of *R. kuepferi*. The relevés were taken from the Alps vegetation database (Lenoir *et al*., 2012) and contain 8239 plots with size < 125 m^2^ that were sampled at elevations between 1000 and 3400 m a.s.l. and that have a maximum of 5% of tree or tall shrub (> 2 m) cover (see Nieto‐Lugilde *et al*., 2014). These vegetation relevés are georeferenced and include a complete list of vascular plant species. Hence, both full coarse‐grained and fine‐grained environmental data values could be calculated for them in the same way as for our own *R. kuepferi* sampling plots.

Our subsequent comparison of the calculated niches of diploid and tetraploid cytotypes combined several approaches. First, we used the niche equivalency and similarity tests proposed by Warren *et al*. (2008) and implemented in the package ‘ecospat’ for R (Broennimann *et al*., 2014). Both tests rely on the niche overlap metric *D* (Schoener, 1970) which ranges from 0 (no overlap) to 1 (complete overlap). The niche equivalency test is a strict test of niche conservatism. Occurrences of both cytotypes are pooled and randomly re‐split into two data sets, with the respective numbers of occurrences of the two cytotypes kept constant. Niches are considered non‐equivalent if the observed *D* falls within the lower 5% quantile of 100 reshuffled *D*s. By contrast, the niche similarity test examines whether the environmental niche of one cytotype is more (dis)similar to the niche of the other one than expected by chance. For this purpose, a number of points equal to the occurrences of one cytotype is selected at random from the background area; the niche of this random sample is then compared to the observed niche of the other cytotype by means of *D*. This process is repeated 100 times. If the observed *D* is within the upper or lower 2.5% quantile of the resampled *D*s the cytotypes are said to be more similar or more dissimilar, respectively, than expected by chance. The test is repeated in both directions, i.e. by resampling first the occurrences of tetraploids and then those of diploids.

While *D* offers a metric of niche conservatism versus differentiation it contains little information about how the niches of the two cytotypes differ. In particular, it does not differentiate between changes in niche optima and breadths (Glennon *et al*., 2014) and it does not indicate which environmental gradients are responsible for potential differentiation. For comparing niches in terms of optima and breadths, we additionally bootstrapped niche calculations by re‐sampling occurrence points of both cytotypes 100 times. For each re‐sample, we randomly selected 100 cells of the gridded PCA space, with the probability of selection weighted by the cytotypes' occurrence density. From the 100 cells we calculated the niche optimum and the niche breadth as the median and the distances between the 2.5% and 97.5% quantiles along the two PCA axes respectively (cf. Theodoridis *et al*., 2013). We then subtracted, for each pair of re‐samples, the calculated niche optima and niche breadths for the tetraploid cytotype from those of the diploid cytotype. If the 95% confidence interval of these differences did not include 0, we considered the niche optima and niche breadths to be different respectively. Moreover, we calculated the proportions of the niche of the tetraploid cytotype which is not occupied by the diploid cytotype and vice versa (cf. Petitpierre *et al*., 2012; Guisan *et al*., 2014). Finally, we plotted the direct overlay between the two niches in PCA space for visual inspection of the environmental drivers responsible for the detected niche changes. All analyses were conducted in R version 3.1.2 (R Core Team, 2014).

### Set of comparisons

Apart from using the total set of sampled populations to compare the niches of diploid and tetraploid cytotypes of *R. kuepferi* across the full Alpine range of the species, we further conducted separate comparisons among differently defined subsets of our 102 populations. In particular, (1) we restricted our comparison to the populations in the south‐western part of the Alps where the two cytotypes are sympatric; (2) by contrast, we compared diploid populations to tetraploid populations sampled outside the sympatric area, i.e. we compared niches of allopatric diploid and tetraploid populations. Finally, (3) we compared the tetraploid populations in the south‐western Alps with the tetraploid populations in the rest of the Alps, i.e. allopatric tetraploid populations. All these comparisons were conducted based on both the coarse‐ and the fine‐grained environmental variable sets, with the background areas adjusted to the ranges of the populations to compare. We also ran an alternative analysis using the total range of the species as a background area for all comparisons which delivered very similar results (see Appendix S2). Mixed populations, i.e. those that contained both diploid and tetraploid individuals (or tri‐ and pentaploids) were excluded from the analyses because tetraploid individuals in diploid populations may only represent a transient phenomenon.

## Results

**Figure 2:**
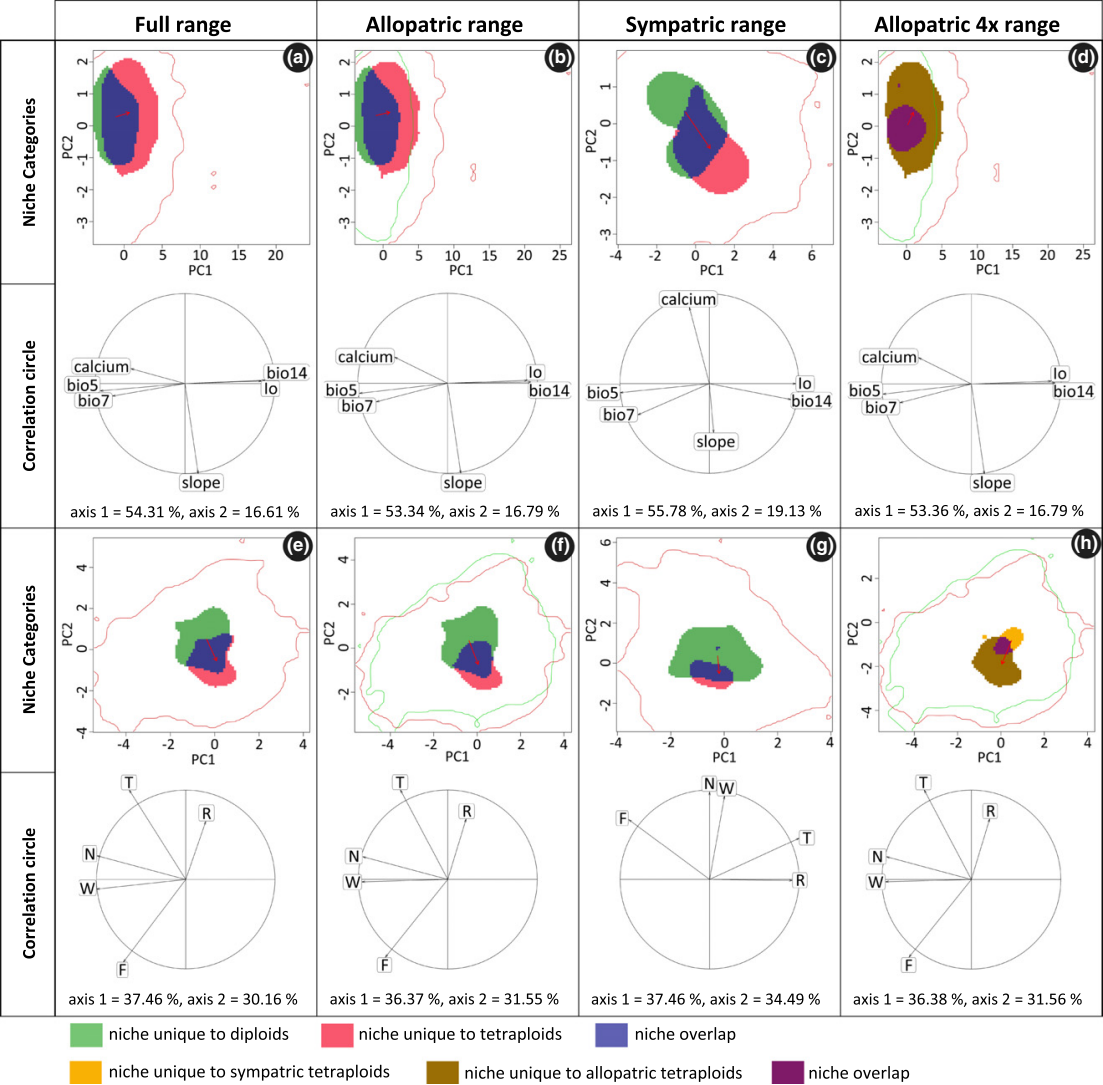
Niche change observed with coarse‐grained environmental (a–d) and fine‐grained environmental (e–h) variables comparing diploid and tetraploid *Ranunculus kuepferi* populations in their full (a, e), allopatric (b, f) and sympatric (c, g) range and comparing tetraploid *Ranunculus kuepferi* populations within the sympatric and outside the sympatric area (d, h). Area of niche unique to diploids, niche overlap and niche unique to tetraploids (a–c, e–g) are shown in green, blue and red respectively. Area of niche unique to tetraploids in the sympatric area, niche overlap and niche unique to tetraploids outside the sympatric area (d, h) are shown in orange, purple and brown respectively. The red arrow links the centroid of the diploids and tetraploids niche (a–c, e–g) and tetraploids niche in the sympatric and outside the sympatric area (d, h) respectively. The available environment in the study area(s) are defined by red lines when comparing populations with the same background area (a, c, e, g) and by green and red lines when comparing populations from the sympatric with populations outside the sympatric area (b, d, f, h). The correlation circle shows the loadings of individual environmental variables to the two PCA axes. bio5: maximum temperature of warmest month; bio7: annual temperature range; bio14: precipitation of driest month; Io: ombrothermic index; calcium: percentage of calcareous soils; slope: slope inclination; *T*: temperature, F: average soil moisture during the growing season; W: variability of soil moisture during the growing season; R: soil pH; N: soil nutrient content. T, F, W, R, N are mean Landolt indicator values for the communities occupying the sampling plots.

Of the 102 populations sampled, 23 were purely diploid and 60 purely tetraploid. From the 60 tetraploid populations, seven were sampled within and 53 outside the range of the diploids. The remaining 19 populations, all from the contact zone in the south‐western Alps, either contained diploid, triploid and tetraploids (8 populations), or diploids and triploids (2), or triploids, tetraploids and pentaploids (9) (Fig. 1). Based on the complete set of all 83 populations, the coarse‐ and fine‐grain environmental analyses revealed largely consistent results: Schoener's *D*‐values indicate that the niches of the diploids and tetraploids are not equivalent and are not more similar than expected by chance (Table 1). In particular, niches have different optima, either on the first or on both PCA axes, with the coarse‐ and fine‐grained environmental variables (*P *<* *0.001, *P *=* *0.230; and *P *=* *0.01, *P *<* *0.001) respectively. By contrast, niche breadths do not differ significantly among the two cytotypes. Only with respect to the niche space unique to diploids/tetraploids, respectively, do the two variable sets deliver contradictory results (Table 1, Fig. 2a,e): while the coarse‐grain variables suggest that tetraploids still occupy most of the diploid niche but have also expanded considerably beyond (cf. Table 1, net expansion* *=* *0.231), the fine‐grained data indicate that the new niche space captured by tetraploids is smaller than the diploid niche space they do not occupy anymore (cf. Table 1, net expansion* *=* *−0.286, i.e. a net loss of niche space).

**Table 1:** Results from the niche overlap metric (Schoener's *D*), niche equivalency and the two niche similarity tests, and from the comparison of changes of niche optima and niche breadth between diploid and tetraploid populations of *Ranunculus kuepferi* (full, allopatric and sympatric range) and between tetraploid populations within and outside the sympatric area (allopatric 4× range). ‘Expansion versus unfilling’ is the subtraction of niche parts unique to diploids from niche parts unique to tetraploids (full, allopatric and sympatric range) and niche parts unique to tetraploids within the sympatric range from niche parts unique to tetraploids outside the sympatric area (allopatric 4× range); positive values indicate that tetraploids (or tetraploids outside the sympatric area) have expanded, and negative values indicate that they have unfilled (parts of) the diploids' (or the sympatric tetraploids') niche respectively. Significant *P*‐values (< 0.05) are shown in bold. Niche broadening is symbolized by >, niche contraction by <. Values for ‘Schoener's *D*’ and ‘expansion versus unfilling’ are not *P*‐values

	Full range		Allopatric range		Sympatric range		Allopatric 4× range	
	Coarse‐grain	Fine‐grain	Coarse‐grain	Fine‐grain	Coarse‐grain	Fine‐grain	Coarse‐grain	Fine‐grain
Schoener's *D*	0.278	0.235	0.332	0.270	0.263	0.098	0.222	0.112
Equivalency	**0.020**	**0.020**	**0.022**	**0.021**	0.081	0.020	0.104	**0.027**
Similarity 1 → 2	0.438	0.288	0.336	0.254	0.226	0.325	0.323	0.299
Similarity 2 → 1	0.461	0.351	0.342	0.207	0.172	0.317	0.072	0.234
Niche optimum PC1	**0.000**	**0.010**	**0.000**	**0.000**	**0.000**	0.390	**0.000**	**0.030**
Niche optimum PC2	0.230	**0.000**	0.290	**0.000**	**0.030**	**0.000**	0.070	**0.000**
Niche breadth PC1	0.140>	0.110<	0.160>	0.050<	0.140<	**0.000<**	**0.020>**	0.260>
Niche breadth PC2	0.110>	0.060<	0.150>	**0.030<**	0.160<	**0.020<**	**0.000>**	**0.000>**
Expansion versus unfilling	0.231	−0.286	0.197	−0.375	−0.066	−0.629	0.575	0.309

When restricting the comparison to allopatric diploid and tetraploid populations, the results are the same: both data sets suggest shifts in the niche optima but they disagree with respect to net niche expansion (coarse‐grained environmental variables: net expansion: 0.197) or reduction (fine‐grained environmental variables: net expansion: −0.375) of the tetraploids’ niche respectively. Other than with the total set of populations, however, the large niche space unique to diploids in the fine‐grained analysis also translated into a statistically significant reduction in the tetraploids’ niche breadth (PCA1: *P *=* *0.05, PCA2: *P *=* *0.03).

Within the sympatric area, differences among coarse‐ and fine‐grained environmental analyses are more pronounced (Table 1; Fig. 2c,g). With coarse‐grained data, the results are similar to those obtained for full range and allopatric range analyses (Table 1). There is a significant shift of the niche optimum on both PCA axes (*P *<* *0.001), but no change in niche breadth. Consequently, the parts of the niches unique to diploids and tetraploids, respectively, are approximately balanced (net expansion: −0.066). With the fine‐grain data, *D‐*values are considerably lower (coarse‐grain: 0.263, fine‐grain: 0.098) indicating higher dissimilarity, with niche optima differing less, but niche breadths differing much more. In particular, the niches of tetraploids appear to be much narrower with this restriction being mainly driven by a large part of the diploid niche space not occupied by tetraploids (net expansion: −0.629).

Finally, the comparison of allopatric tetraploids revealed that tetraploids outside the south‐western Alps have shifted their niche optima compared to tetraploid populations that co‐occur with diploids; and they have significantly broader niches, mainly due to a considerable niche expansion (Table 1, Fig. 2d,h). These trends are consistent at both coarse‐ and fine‐grained resolutions.

With respect to the main environmental axes of niche differentiation for the full and allopatric ranges, coarse‐grained environmental variables indicated that tetraploids have shifted their niche optima along the first PCA axis (optima shifts are indicated by red arrows in Fig. 2), i.e. towards cooler summer temperatures (bio5), less pronounced differences among summer and winter temperatures (bio7), less calcareous soils (calcium), but higher precipitation during the driest month (bio14) and a higher ombrothermic index (Io) (Fig. 2a,b; see also Appendix S3). Fine‐grain environmental results are similar but suggest that niches are most distinct along the temperature gradient (T) (Fig. 2e,f). In the sympatric range, coarse‐grained environmental predictors indicate an approximately equal contribution of almost all variables to the shifting niche optimum (Fig. 2c). By contrast, with fine‐grained data, optima in the sympatric range differ mainly in nutrient availability (N) and annual soil moisture variability (W, Fig. 2c). Complementarily, when comparing allopatric ranges of tetraploids, both the coarse‐ and fine‐grained variables suggest that outside the south‐western Alps tetraploids have expanded their niches with respect to more or less all environmental gradients, and in particular to cooler and moister conditions as well as towards more acidic soils (Fig. 2d,h).

## Discussion

Overall, our results demonstrate that diploid and tetraploid populations of *R. kuepferi* differ in terms of niche optima and breadths, and that tetraploids have occupied new niche space while leaving part of the diploid niche space unfilled. The magnitude and nature of these differences depends, however, on whether cytotypes are compared in sympatric or allopatric situations as well as on the spatial grain of the environmental variables used to characterize the cytotypes’ niches. When focussing on the entire ranges of both cytotypes, niche differentiation appears to be mainly driven by a shift of the niche optimum. Analysing the subset of sympatric and allopatric populations separately and at a fine grain indicates, however, that sympatric cytotypes are characterized less by a shift of the optimum, but rather by a considerable decrease in the tetraploids’ niche breadth. Hence, the major change in the niche optimum, together with a moderate re‐expansion of niche breadth, apparently occurred only after the tetraploids had migrated out of the sympatric range, or during this migration process.

### Differences among coarse‐ and fine‐grain data sets

The partly inconsistent results achieved with coarse‐ and fine‐grained environmental variables may theoretically result from the fact that we did not use exactly the same variables at both scales. However, we do not think that differences in variable sets were the main reason for the detected inconsistencies because, first, we used fine‐scale indicator values that should, with the exception of N (nutrient availability), represent the same physical gradients as captured by the broad‐scale variables (temperature, water availability, and soil pH); and, second, because shifts in optima are mostly congruent between scales (for example, a shift towards lower mean temperature of the warmest month at the broad scale is paralleled by a shift towards a lower *T* indicator value), whereas changes in niche breadths consistently differ between them. In general, analyses with fine‐grained data indicated that niches of tetraploids are narrower than those of diploids while with coarse‐grained variables niche breadth does not differ, or alternatively the results even indicate net niche expansion of tetraploids. These differences suggest that the coarse‐scale analysis overestimates the environmental tolerance of tetraploid *R. kuepferi*. We assume that the higher variation in coarse‐scale climatic conditions associated with the larger geographical range of the tetraploids is mainly responsible for this overestimation. The fine‐scale analysis, in comparison, suggests that the tetraploids respond to changes in coarse‐scale climate conditions by selecting microsites that keep physical conditions more constant than they appear at the coarser scale. In rugged high‐mountain terrain, there are ample opportunities for such microsite selection as both climatic and non‐climatic conditions can vary greatly over short distances (e.g. Scherrer & Körner, 2010). This does not imply that cytotypes of mountain species will always differ when analysed at an ever finer scale (see e.g. Baack & Stanton, 2005). Nevertheless, these results suggest that niche differentiation of mountain species based on coarse‐scale climatic maps such as those derived from WorldClim may easily produce misleading results, in particular with respect to changes in niche breadths.

### Niche differentiation among diploids and tetraploids

In agreement with recent studies of other European mountain species (e.g. Sonnleitner *et al*., 2010; Theodoridis *et al*., 2013), our results suggest that the realized niche optimum of tetraploids has shifted relative to their diploid ancestors. In particular, tetraploids occur in cooler climates and under more acid and less nutrient‐rich conditions. Such a shift is consistent with the common assumption that polyploids are able to colonize harsher environments than their diploid ancestors (e.g. Brochmann *et al*., 2004; Treier *et al*., 2009). In Alpine species, a possible reason for this phenomenon is that polyploidization has resulted in a diverse array of novel genetic combinations that conferred polyploids an advantage when colonizing formerly glaciated terrain during interglacials (e.g. Kearney, 2005). The argument is particularly appealing where hybridization is involved in polyploid formation (Comai, 2005). *R. kuepferi* is autotetraploid (Cosendai *et al*., 2011), but the polyploids combine the geographically differentiated gene pools of the diploid source populations (Cosendai *et al*., 2013) and hence represent a case of intraspecific hybrids. In addition, or even more importantly, we hypothesize that the change of reproductive systems will have facilitated the establishment of tetraploid *R. kuepferi* in environments cooler than those tolerated by diploid ancestors: pseudogamous apomixis (embryo formation without fertilization, but the endosperm can develop only after fertilization) combined with self‐pollination implies reproductive assurance if mating partners and/or pollinators are rare, as is often the case in high‐mountain environments (Bergman *et al*., 1996). Actually, a change from obligate outcrossing to selfing has also been postulated as an important factor for the initial establishment and subsequent niche differentiation of the polyploid Alpine species *Primula halleri* J.F.Gmel (Theodoridis *et al*., 2013).

Polyploids are often assumed to have niches which do not only differ in optima, but which are also broader than those of their diploid ancestors (e.g. Levin, 1983; Otto & Whitton, 2000). However, empirical support for this hypothesis is mixed (e.g. Martin & Husband, 2009; Treier *et al*., 2009; McIntyre, 2012). In particular, Theodoridis *et al*. (2013) have recently demonstrated that niche breadth is actually smaller in polyploids of the *Primula* sect. *Aleuritia* complex in the Alps and other European mountain ranges. These results are consistent with our own findings for *R. kuepferi* and underline that differences in niche breadth are probably an idiosyncratic feature of heteroploid cytotypes which may, among other factors, depend on the time available to expand niche breadths. In the case of *R. kuepferi* we hypothesize that the narrower niche of tetraploids might, again, be a consequence of the change in the reproductive system towards apomixis. Asexual reproduction implies clonal freezing of apomictic lineages (cf. Vrijenhoek & Parker, 2009). If these lineages do not cover the full range of the ancestor's niche this mechanism may cause niche restriction because further local adaptation by recombination is impossible or will occur at lower rates in the case of facultative apomicts. In *R. kuepferi*, it seems that tetraploid lineages at the warm margins of the diploid niche have never established in the sympatric range, and that during geographical expansion tetraploids have been able to progressively adapt to cooler conditions but not to re‐fill the warmer part of the diploids’ niche. At least indirectly, the narrower niche of the tetraploids might hence be an effect of competition (below).

### The role of niche differentiation for polyploid establishment

Niche differentiation is considered a prerequisite of polyploid establishment under the double challenge of a mating disadvantage (minority cytotype exclusion, Levin, 1975) and of resource competition with a closely related species (e.g. Theodoridis *et al*., 2013). In *R. kuepferi*, the problem of minority cytotype exclusion has obviously been ‘solved’ – and the need for niche differentiation hence relaxed – by a switch to apomixis. The detected differences between the two cytotypes’ niches may hence rather be a response of tetraploids to resource competition; or they evolved independent of interactions with diploids and were hence not a prerequisite but a byproduct of cytotype differentiation. There are two aspects of our results to consider in this context. On the one hand, the strong reduction in tetraploids to a marginal part of the diploid niche in the sympatric range suggests that diploids might actually restrict the distribution of tetraploids where both cytotypes co‐occur. This might be surprising as polyploidization is often associated with increased vigour, so‐called heterosis (Comai, 2005). However, population growth rates of polyploids are not necessarily higher than those of diploids (Münzbergova, 2007) and in the specific case of *R. kuepferi*, seed set data of the investigated populations have shown that diploids are superior to tetraploids in terms of reproductive outputs (Schinkel *et al*., submitted). In addition, the fact that tetraploid lineages have not established in the warm part of the diploid niche, neither in the sympatric nor in the allopatric range, is also consistent with the assumption that tetraploids are weaker competitors than diploids in *R. kuepferi* because competitive intensity among plants generally increases under less stressful, in this case warmer conditions (e.g. Bertness & Callaway, 1994; Callaway *et al*., 2002). On the other hand, the most important shift of the tetraploid's niche optimum, the one towards cooler conditions, was fully realized only after the cytotypes have become spatially segregated and was hence obviously not a response to competition with the ancestor. Taken together, these results suggest that competition with diploids does constrain the distribution of tetraploids but is not effective enough to exclude tetraploids from the sympatric range although the niches of the two cytotypes nearly completely overlap there. Niche differentiation was hence probably no prerequisite of polyploid establishment. However, as discussed above, restriction to the marginal parts of the diploid niche in the sympatric range may have directed the further evolution of the tetraploids’ niche towards cooler conditions in the allopatric range.

If tetraploids are actually competitively inferior to their diploid ancestors, why then have they been so much more successful in re‐colonizing the Alps after the Last Glacial Maximum? The progressively acquired physiological tolerance of cooler environments might have played a certain role as the diploids’ range at the south‐western margins has a slightly warmer climate than the rest of the Alps. However, we hypothesize that, again, the change in the reproductive system from sexuality to apomixis has been a powerful driver of tetraploid spread. Ability to reproduce without mates or pollinators can be a key advantage in colonizing new areas (‘Baker's Law’, Baker, 1967) because a new population can establish from a single propagule. In addition, polyploid apomicts, which do not undergo recombination in the reproduction process, may maintain high levels of heterozygosity, a result of the polyploid origin, and hence reduce detrimental founder effects (Hörandl, 2006; Cosendai *et al*., 2011, 2013).

## Conclusions

Our results demonstrate that across their full ranges, niches of diploid and polyploid cytotypes of *R. kuepferi* differ in their optima, with a shift towards cooler conditions in tetraploids as the most important distinction. However, this shift was probably a secondary phenomenon which developed during, or after expansion of the tetraploids' geographical range. Niche differentiation was hence probably not a prerequisite of tetraploid establishment. Analysing sympatric and allopatric populations separately thus helped us to avoid misleading conclusions and to provide a more realistic insight into the processes involved in cytotype establishment.

Rather than niche differentiation, we assume that the change in the reproductive system was decisive for the initial establishment of *R. kuepferi*. Given that resource competition among plants is commonly diffuse rather than species‐specific, avoidance of minority cytotype exclusion might often be more important for polyploid persistence than differentiation of abiotic niches, and, in case of *R. kuepferi*, the switch to apomixis was certainly effective in avoiding such minority cytotype exclusion.

Finally, the comparison of analyses based on coarse‐ and fine‐grained data revealed that the former tend to overestimate niche breadths of the more widely distributed taxon and we thus strongly recommend basing inferences from niche comparison analyses on environmental data computed at a grain size that is appropriate for the organism and question under study (Potter *et al*., 2013).

## Biosketch

**Bernhard Kirchheimer** is a PhD student at the Department of Botany and Biodiversity Research of the University of Vienna in Austria. He is interested in plant community ecology, in particular in understanding patterns and drivers of plant species' distributions.

Author contributions: S.D., E.H. and B.K. conceived the ideas. B.K., C.C.F.S., A.D., S.K., M.W., E.H. and S.D. collected field data. J.L., M.C., A.G., J.‐C.S., W.T., P.V., W.W., N.E.Z. provided the relevé data. C.C.F.S. determined ploidy level. D.M. prepared the GIS data. B.K. analysed the data and B.K. and S.D. led writing. All authors discussed results and commented on the text.

## Supporting information


**Appendix S1** Sources of occurrence records, data preparation and downscaling procedure of climate data.

**Appendix S2** Alternative analysis using the total range of the species as a background area for all comparisons.

**Appendix S3** Kernel density plots of each environmental variable used for the analysis.
