# Supplementary material for: A matter of scale: apparent niche differentiation of diploid and tetraploid plants may depend on extent and grain of analysis
Source: J Biogeogr. 2015 Dec 11;43(4):716–26. doi: 10.1111/jbi.12663 (PMC4966631; doi:10.1111/jbi.12663)
Supplement: Supplementary file 1 — Appendix S1 Sources of occurrence records, data preparation and downscaling procedure of climate data. [file JBI-43-716-s001.doc]

*Journal of Biogeography*

**Supporting Information**

**A matter of scale: apparent niche differentiation of diploid and tetraploid plants may depend on extent and grain of analysis**

Bernhard Kirchheimer, Christoph C. F. Schinkel, Agnes S. Dellinger, Simone Klatt, Dietmar Moser, Manuela Winkler, Jonathan Lenoir, Marco Caccianiga, Antoine Guisan, Diego Nieto-Lugilde, Jens-Christian Svenning, Wilfried Thuiller, Pascal Vittoz, Wolfgang Willner, Niklaus E. Zimmermann, Elvira Hörandl, Stefan Dullinger

**Appendix S1:** Sources of occurrence records and downscaling procedure of climatic data.

*Occurrence records*

The following sources have been searched for occurrence records of *Ranunculus kuepferi*: private herbarium of Philippe Küpfer, herbaria NEU (University of Neuchatel; Switzerland), GOET (University of Göttingen; Germany), W (Natural History Museum Vienna; Austria), GZU (Karl Franzens University of Graz; Austria), WU (University of Vienna; Austria), Z (University of Zürich; Switzerland), KL (Herbarium of Carinthia; Austria), the Global Biodiversity Information Facility (GBIF, [www.gbif.org](http://www.gbif.org/), last access 2014-05-13), as well as the free internet, e.g. “flickr” ([www.flickr.com](http://www.flickr.com/), last access 2014-05-13).

*Data preparation and downscaling procedure of climatic data*

Climatic data used for the coarse-grained environmental data was derived from WorldClim, which provides monthly averages of mean, minimum and maximum temperature as well as monthly precipitation sums for the period of 1950 – 2000, together with 19 bioclimatic variables which are derived from the monthly values at a spatial resolution of 30 arc-seconds (approximately 1km at the equator). These data were statistically downscaled to a resolution of 100 x 100 m to match the size of our sampling plots.

The downscaling procedure can be summarized as follows: at the 1 km spatial resolution, we analysed the dependency of precipitation and temperature on elevation by means of linear regressions in circular moving windows of 15 and 25 km radius, respectively. We chose smaller moving windows for precipitation, because of the better fit in cross-validation exercises. By doing so, we extracted the hidden lapse rates and ’0 m above sea level‘ temperature and precipitation intercepts inherent in the WorldClim maps. We stored lapse rates and intercepts to the centre cell of each window position and then spatially interpolated these regression parameters to a 100 m resolution by means of inverse distance-weighted interpolation. Finally, the interpolated regression parameters were applied for back conversion to climate maps using a 100 m digital elevation model, which was aggregated from the 90 m SRTM DEM (Rabus *et al*., 2003) version 4.0 by means of the AGGREGATE command in ArcGrid. In summary, this procedure allowed us to first extract the hidden regression parameters of the WorldClim maps, and then to spatially scale them to the resolution of 100 m. The same approach to statistical downscaling of climatic parameters has already been used in several earlier studies (Zimmermann *et al*., 2007, 2009; Randin *et al*., 2009; Engler *et al*., 2011; Dullinger *et al*., 2012).

To better represent water availability during the vegetation period we additionally calculated an ombrothermic index (Io) from these climatic data as Io = 10(YPP/YPT), where YPP is the yearly positive precipitation (i.e., summed precipitation of the months with an average temperature > 0°C) and YPT is the yearly positive temperature (i.e., sum of the average temperature of the months with an average temperature > 0°C) (Rivas-Martinez, 1996 cited in Attorre *et al.*, 2007).

From the array of climatic data we then selected four variables which (1) were not too closely correlated (Pearson *r* < 0.75) among each other to avoid collinearity issues (Dormann *et al.*, 2012) and (2) represent the most important climatic drivers of plant growth: temperature (maximum temperature of warmest month and annual temperature range) and water availability (precipitation of driest month and ombrothermic index).

On top of these climatic variables we added slope inclination and information on substrate conditions. The former was calculated from a Digital Elevation Model (Jarvis *et al.*, 2008) of 100 m cell size and the latter using information from the European Soil Database (ESDB, http://eusoils.jrc.ec.europa.eu). With the data from the ESDB we calculated the percentage area of Soil Typological Units (STU) having a calcareous dominant parent material for every Soil Mapping Unit (SMU). For the Austrian part of the Alps, a fine-scaled map of substrate units was available (Bayer & Pavlik, 2009) which we used to compute the area of calcareous substrates within grid cells of 5’ longitude × 3’ latitude in order to get information compatible to that derived from the European Soil Database. These environmental variables were estimated for each of the 100 x 100 m sample plots by GIS overlay.

**REFERENCES**

Attorre, F., Alfo, M., De Sanctis, M., Francesconi, F. & Bruno, F. (2007) Comparison of interpolation methods for mapping climatic and bioclimatic variables at regional scale. *International Journal of Climatology*, **27**, 1825-1843.

Bayer, K. & Pavlik, W. (2009) Databases at the Geological Survey of Austria: Substrate units in the Austrian Alps at the scale of 1:200.000. 6th European Congress on Regional Geoscientific Cartography and Information Systems (EUREGEO), Earth and Man. Proceedings 1: 109-111. Landesamt für Vermessung und Geoinformation, München.

Dormann, C.F., Schymanski, S.J., Cabral, J., Chuine, I., Graham, C., Hartig, F., Kearney, M., Morin, X., Romermann, C., Schroder, B. & Singer, A. (2012) Correlation and process in species distribution models: bridging a dichotomy. *Journal of Biogeography*, **39**, 2119-2131.

Dullinger, S., Gattringer, A., Thuiller, W. et al. (2012) Extinction debt of high-mountain plants under twenty-first-century climate change. *Nature Climate Change*, 2, 619-622.

Engler R., Randin, C.F., Thuiller, W. et al. (2011) 21st century climate change threatens mountain flora unequally across Europe. *Global Change Biology*, 17, 2330–2341.

Jarvis, A., Reuter, H.I., Nelson, A. & Guevara, E. (2008) Holefilled seamless SRTM data V4. International Centre for Tropical Agriculture (CIAT). Available at: http://srtm.csi.cgiar.org

Rabus, B., Eineder, M., Roth, A. & Bamler, R. (2003) The shuttle radar topography mission - a new class of digital elevation models acquired by spaceborne radar. Isprs Journal of Photogrammetry and Remote Sensing, 57, 241-262.

Randin, C.F., Engler, R., Normand, S., Zappa, M., Zimmermann, N.E., Pearman, P.B., Vittoz, P., Thuiller, W. & Guisan, A. (2009) Climate change and plant distribution: local models predict high-elevation persistence. *Global Change Biology*, **15**, 1557–1569.

Rivas-Martinez, S. (1996) Clasificación Bioclimática de la tierra. *Folia Botanica Matritensis*, **16**, 1–32.

Zimmermann, N.E., Edwards, T.C., Moisen, G.G., Frescino, T.S. & Blackard, J.A. (2007) Remote sensing-based predictors improve distribution models of rare, early successional and broadleaf tree species in Utah. *Journal of Applied Ecology*, **44**, 1057-1067.

Zimmermann, N.E., Yoccoz, N.G., Edwards, T.C., Meier, E.S., Thuiller, W., Guisan, A., Schmatz, D.R. & Pearman, P.B. (2009) Climatic extremes improve predictions of spatial patterns of tree species. *Proceedings of the National Academy of Sciences of the United States of America*, **106**, 19723-19728.
