# Supplementary material for: A matter of scale: apparent niche differentiation of diploid and tetraploid plants may depend on extent and grain of analysis
Source: J Biogeogr. 2015 Dec 11;43(4):716–26. doi: 10.1111/jbi.12663 (PMC4966631; doi:10.1111/jbi.12663)
Supplement: Supplementary file 2 — Appendix S2 Alternative analysis using the total range of the species as a background area for all comparisons. [file JBI-43-716-s002.doc]

*Journal of Biogeography*

**Supporting Information**

**A matter of scale: apparent niche differentiation of diploid and tetraploid plants may depend on extent and grain of analysis**

Bernhard Kirchheimer, Christoph C. F. Schinkel, Agnes S. Dellinger, Simone Klatt, Dietmar Moser, Manuela Winkler, Jonathan Lenoir, Marco Caccianiga, Antoine Guisan, Diego Nieto-Lugilde, Jens-Christian Svenning, Wilfried Thuiller, Pascal Vittoz, Wolfgang Willner, Niklaus E. Zimmermann, Elvira Hörandl, Stefan Dullinger

**Appendix S2:** Alternative analysis using the total range of the species as the background area for all comparisons.

**Figure S1** Niche change observed using the full study area as environmental background for all analysis with coarse-grained environmental (a-d) and fine-grained environmental (e-h) variables comparing diploid and tetraploid *Ranunculus kuepferi* populations in their full (a, e), allopatric (b, f) and sympatric (c, g) range and comparing tetraploid *Ranunculus kuepferi* populations within the sympatric and outside the sympatric area (d, h). Area of niche unique to diploids, niche overlap and niche unique to tetraploids (a-c, e-g) are shown in green, blue and red respectively. Area of niche unique to tetraploids in the sympatric area, niche overlap and niche unique to tetraploids outside the sympatric area (d,h) are shown in orange, purple and brown respectively. The red arrow links the centroid of the diploids and tetraploids niche (a-c, e-g) and tetraploids niche in the sympatric and outside the sympatric area (d, h) respectively. The available environment in the study area is defined by red lines. The correlation circle shows the loadings of individual environmental variables to the two PCA axes. bio5: maximum temperature of warmest month; bio7: annual temperature range; bio14: precipitation of driest month; Io: ombrothermic index; calcium: percentage of calcareous soils; slope: slope inclination; T: temperature, F: average soil moisture during the growing season; W: variability of soil moisture during the growing season; R: soil pH; N: soil nitrogen content. T, F, W, R, N are mean Landolt indicator values for the communities occupying the sampling plots.

**Table S1** Results from the niche overlap metric (Schoener’s *D*), niche equivalency and the two niche similarity tests and from the comparison of changes of niche optima and niche breadth between diploid and tetraploid populations of *Ranunculus kuepferi* (full, allopatric and sympatric range) and between tetraploid populations within and outside the sympatric area (allopatric 4x range) using the full study area as environmental background. ‘Expansion versus unfilling’ is the subtraction of niche parts unique to diploids from niche parts unique to tetraploids (full, allopatric and sympatric range) and niche parts unique to tetraploids within the sympatric range from niche parts unique to tetraploids outside the sympatric area (allopatric 4x range); positive values indicate that tetraploids (or tetraploids outside the sympatric area) have expanded, and negative values indicate that they have unfilled (parts of) the diploids’ (or the sympatric tetraploids’) niche, respectively. Significant *P*-values (< 0.05) are shown in bold. Niche broadening is symbolized by >, niche contraction by <. Values for ‘Schoener’s *D*’ and ‘Expansion versus unfilling’ are not *P*-values and therefore separated in the table by dotted lines.

**Full range Allopatric range Sympatric range Allopatric 4x range**

coarse-grain fine-grain coarse-grain fine-grain coarse-grain fine-grain coarse-grain fine-grain

Schoener's *D* 0.278 0.235 0.232 0.193 0.330 0.105 0.198 0.114

Equivalency **0.020 0.020 0.020 0.020** 0.088 **0.020** 0.095 **0.026**

Similarity 1 → 2 0.438 0.288 0.463 0.307 0.211 0.208 0.361 0.279

Similarity 2 → 1 0.461 0.351 0.507 0.372 0.179 0.230 0.244 0.295

Niche optimum PC1 **0.000 0.010 0.000 0.000 0.000 0.030** **0.000** 0.050

Niche optimum PC2 0.230 **0.000** 0.190 **0.000** 0.380 0.298 0.180 **0.000**

Niche breadth PC1 0.140 > 0.110 < 0.170 > 0.060 < 0.200 < **0.015 < 0.040 >** 0.150 >

Niche breadth PC2 0.110 > 0.060 < 0.080 > **0.020 <** 0.060 < **0.000 < 0.000 > 0.000 >**

Expansion versus unfilling 0.231 -0.286 0.249 -0.375 -0.442 -0.753 0.592 0.274
