# Supplementary material for: A matter of scale: apparent niche differentiation of diploid and tetraploid plants may depend on extent and grain of analysis
Source: J Biogeogr. 2015 Dec 11;43(4):716–26. doi: 10.1111/jbi.12663 (PMC4966631; doi:10.1111/jbi.12663)
Supplement: Supplementary file 3 — Appendix S3 Kernel density plots of each environmental variable used for the analysis. [file JBI-43-716-s003.doc]

*Journal of Biogeography*

**Supporting Information**

**A matter of scale: apparent niche differentiation of diploid and tetraploid plants may depend on extent and grain of analysis**

Bernhard Kirchheimer, Christoph C. F. Schinkel, Agnes S. Dellinger, Simone Klatt, Dietmar Moser, Manuela Winkler, Jonathan Lenoir, Marco Caccianiga, Antoine Guisan, Diego Nieto-Lugilde, Jens-Christian Svenning, Wilfried Thuiller, Pascal Vittoz, Wolfgang Willner, Niklaus E. Zimmermann, Elvira Hörandl, Stefan Dullinger

**Appendix S3:** Kernel density plots of each environmental variable used for the analysis.

| **Coarse-grained environmental variables** | 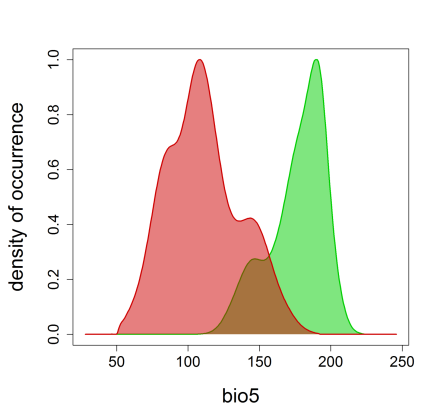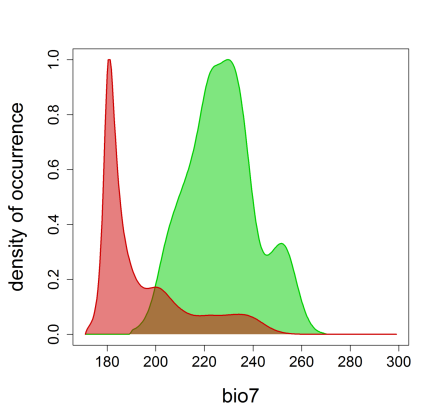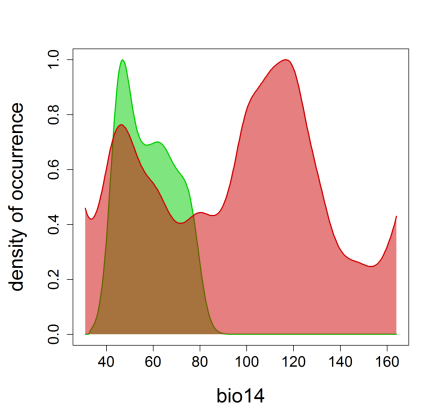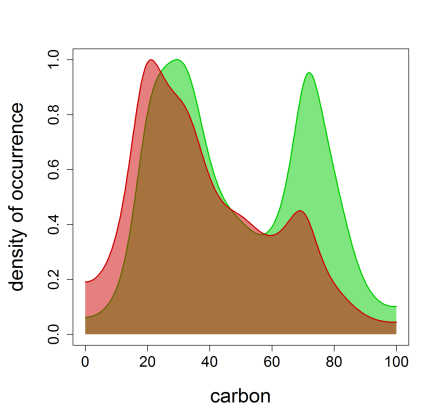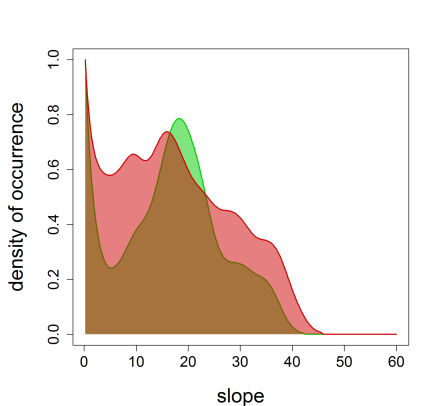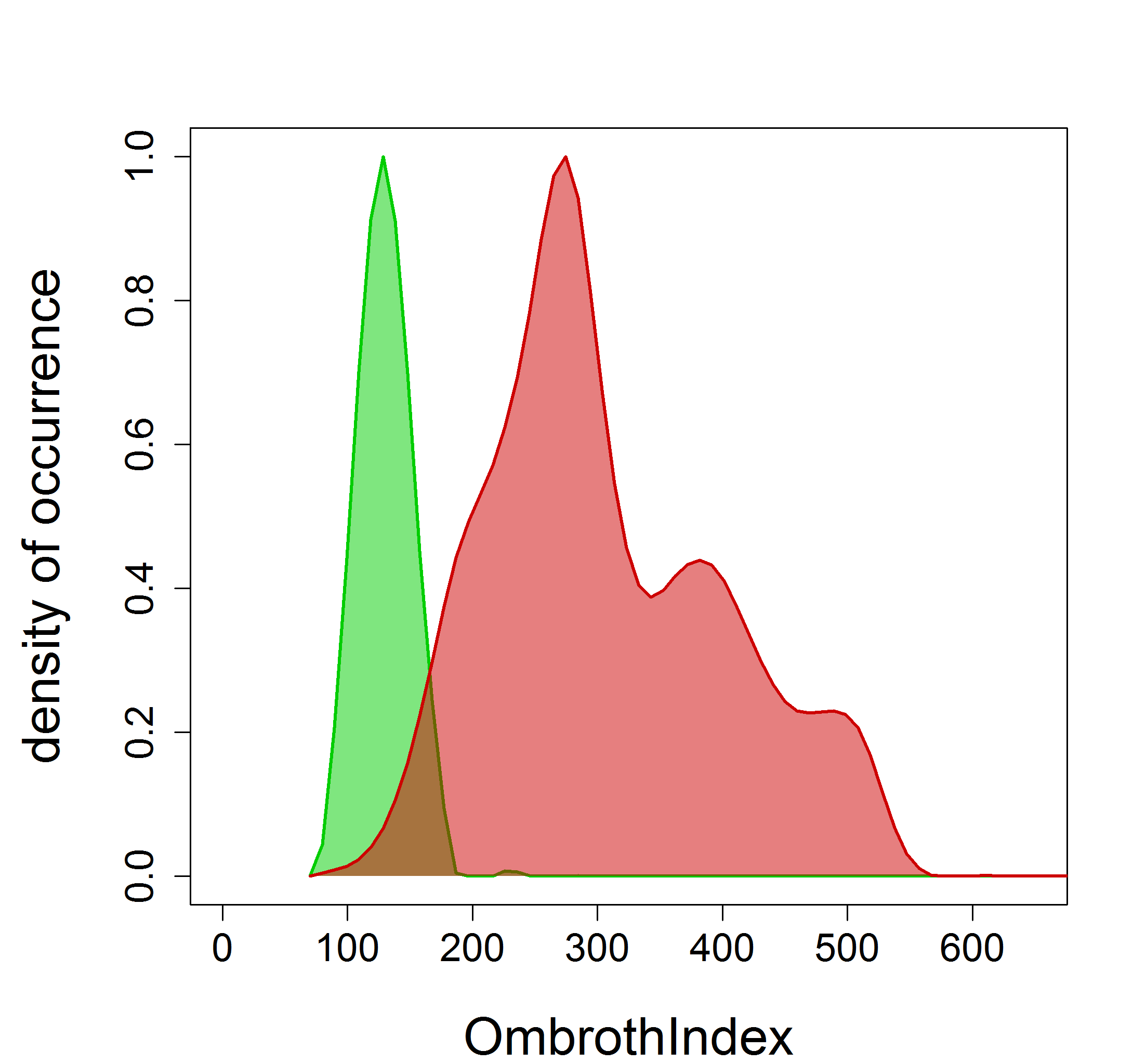 |
| --- | --- |
| **Fine-grained environmental variables** | 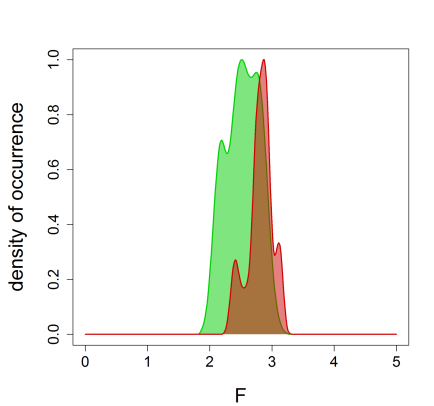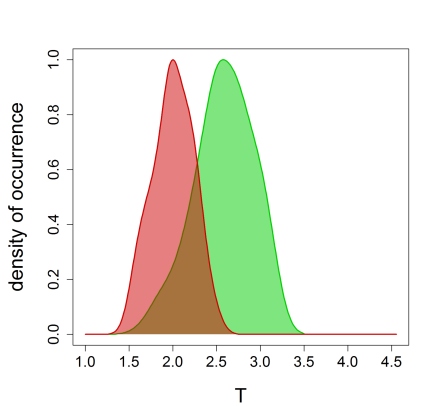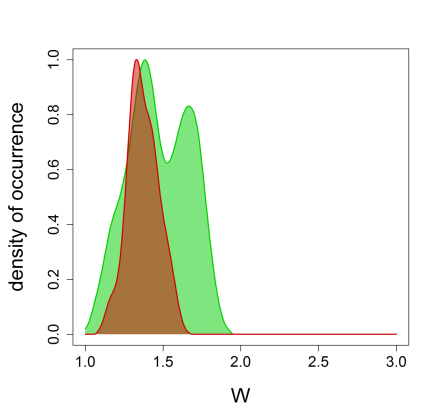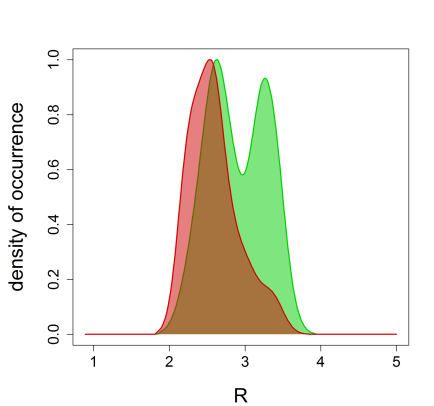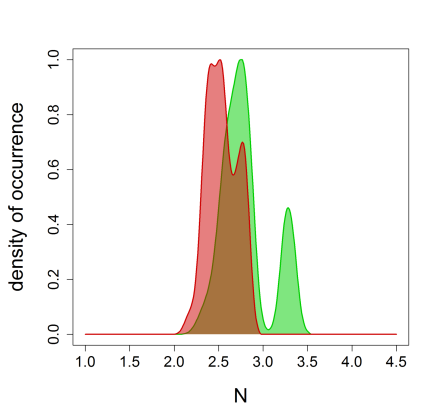 |

**Figure S1** Kernel density plots of the coarse-grained environmental and fine-grained environmental variables comparing diploid and tetraploid *Ranunculus kuepferi* populations in their full range. Densities of diploids and tetraploids are shown in green and red respectively. Measurement units: temperature (°Cx10), precipitation (mm), slope (°), carbon (percentage area of calcareous substrates); for ombrothermic index see Appendix S1; for fine-grained environmental variables see descriptions in main text.

| **Coarse-grained environmental variables** | 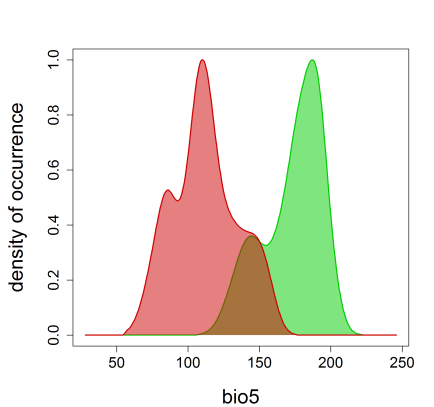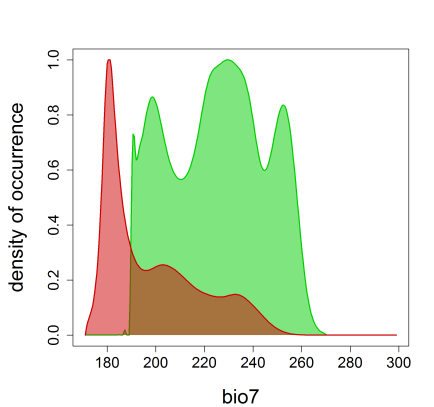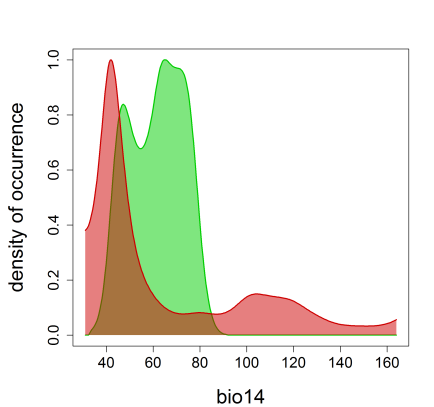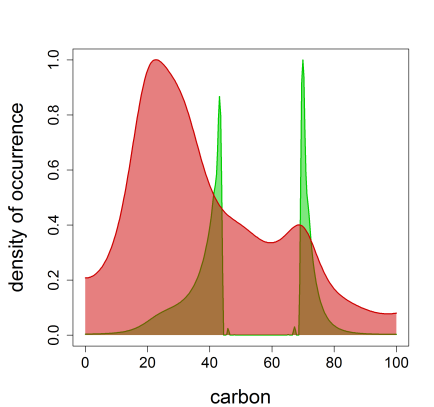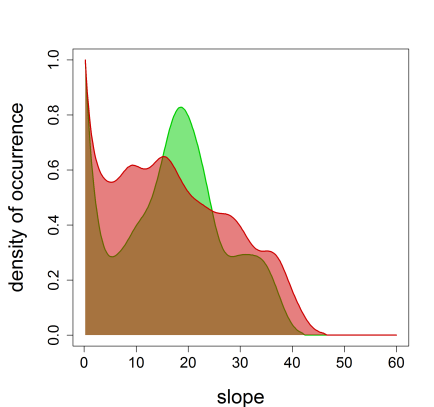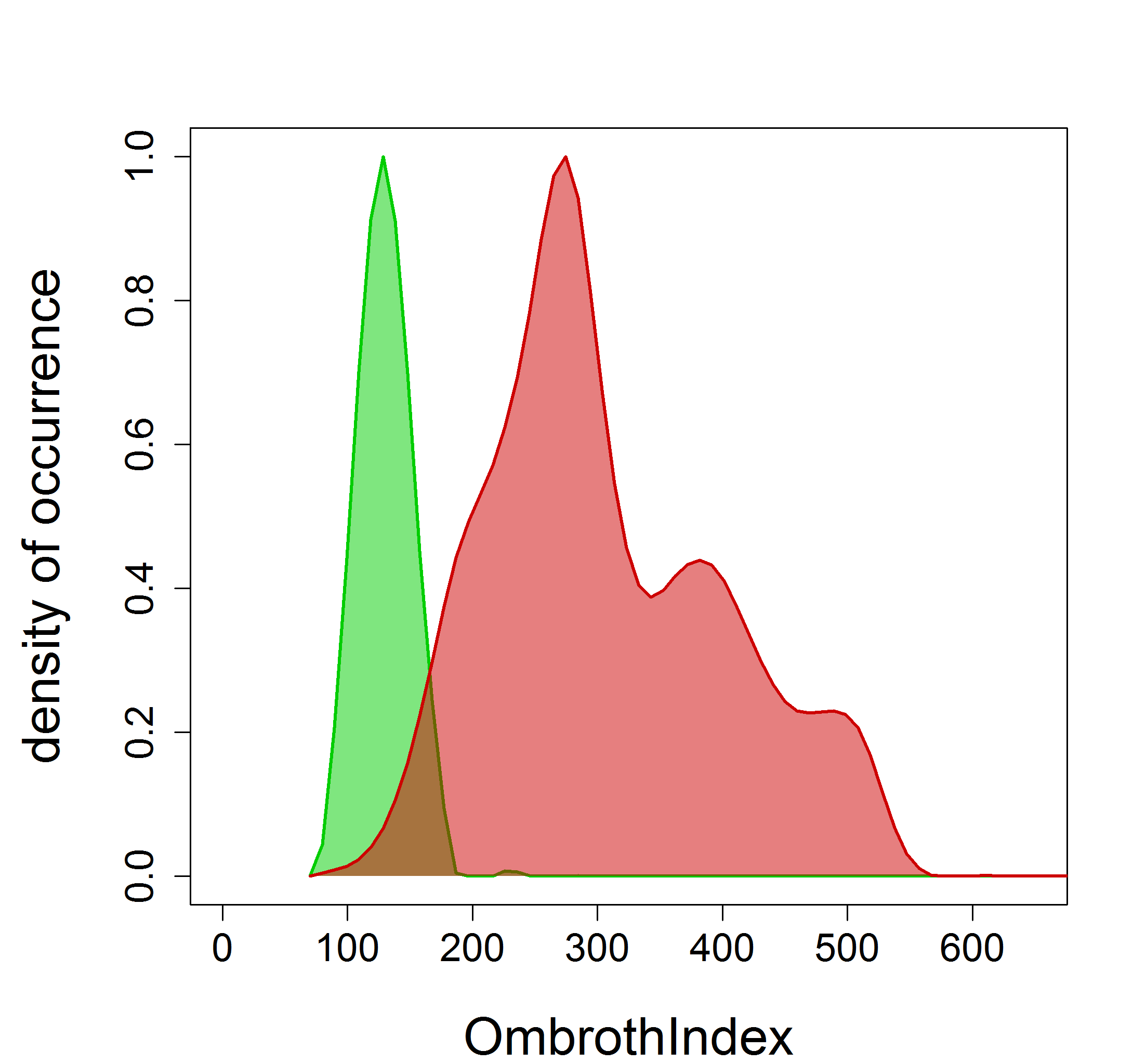 |
| --- | --- |
| **Fine-grained environmental variables** | 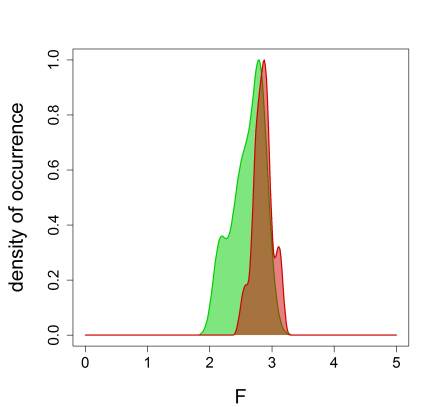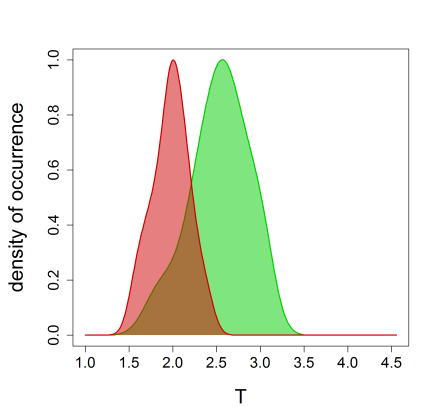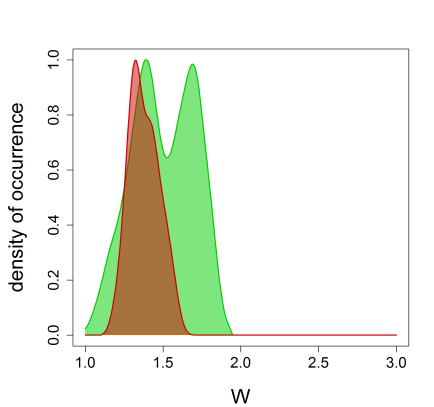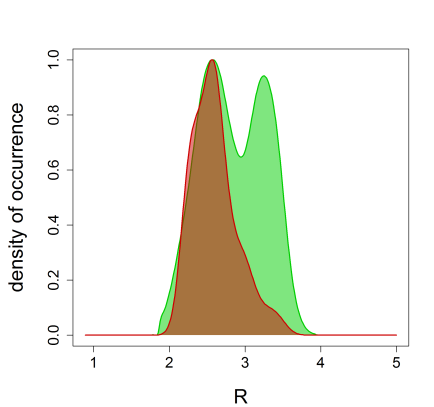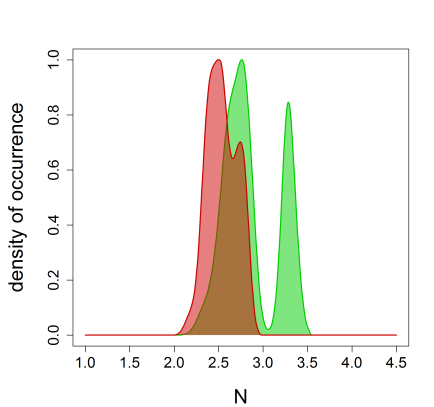 |

**Figure S2** Kernel density plots of the coarse-grained environmental and fine-grained environmental variables comparing diploid and tetraploid *Ranunculus kuepferi* populations in their allopatric range. Densities of diploids and tetraploids are shown in green and red respectively. Measurement units: temperature (°Cx10), precipitation (mm), slope (°), carbon (percentage area of calcareous substrates); for ombrothermic index see Appendix S1; for fine-grained environmental variables see descriptions in main text. The unusual carbon curve is the results of unavailable intermediary conditions in the sympatric area in our data.

| **Coarse-grained environmental variables** | 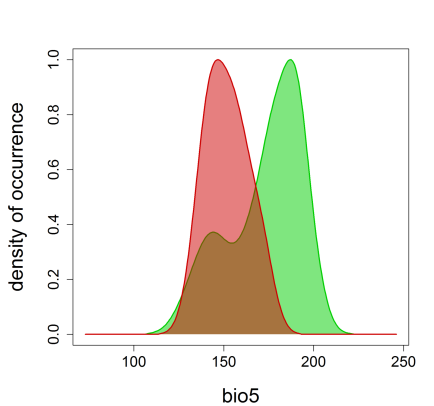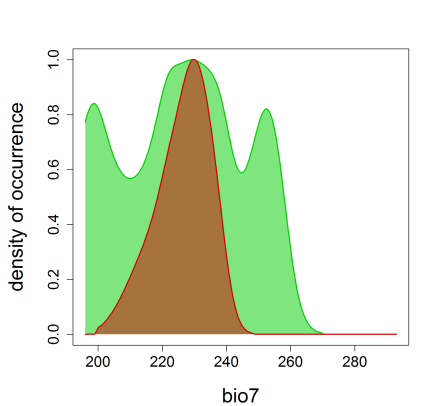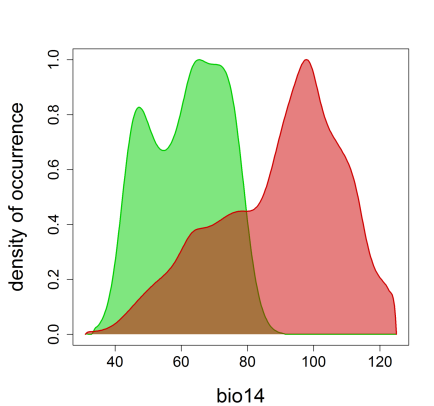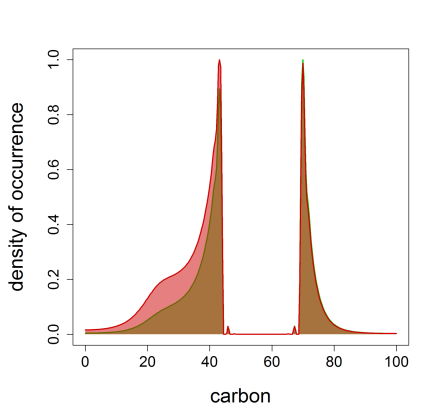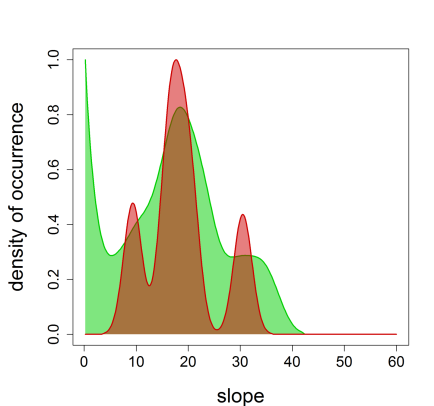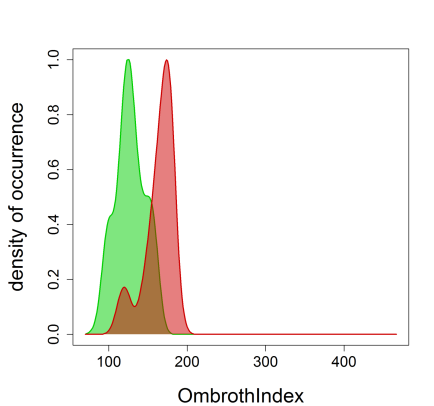 |
| --- | --- |
| **Fine-grained environmental variables** | 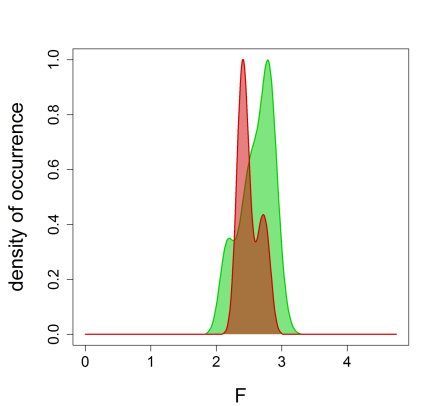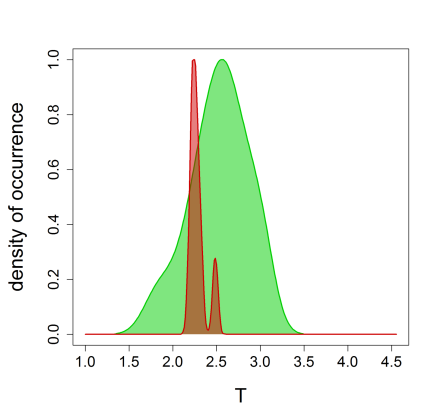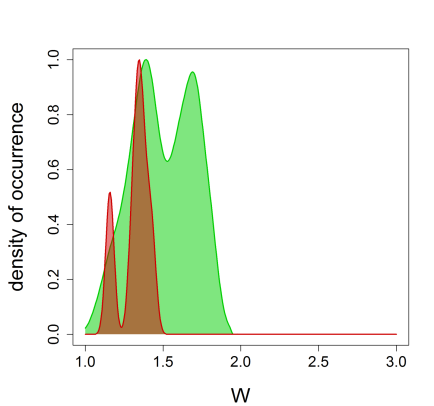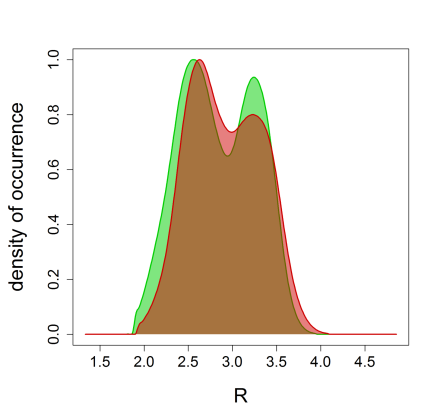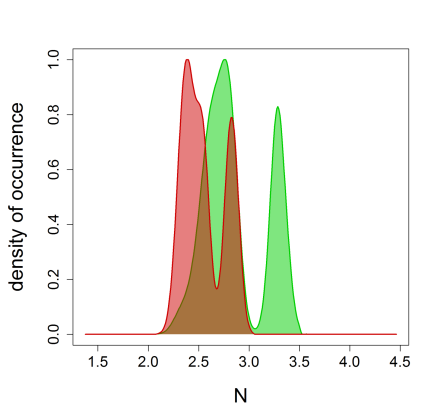 |

**Figure S3** Kernel density plots of the coarse-grained environmental and fine-grained environmental variables comparing diploid and tetraploid *Ranunculus kuepferi* populations in their sympatric range. Densities of diploids and tetraploids are shown in green and red respectively. Measurement units: temperature (°Cx10), precipitation (mm), slope (°), carbon (percentage area of calcareous substrates); for ombrothermic index see Appendix S1; for fine-grained environmental variables see descriptions in main text. The unusual carbon curve is the results of unavailable intermediary conditions in the sympatric area in our data.

| **Coarse-grained environmental variables** | 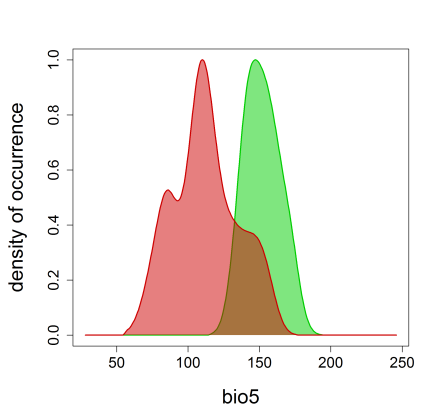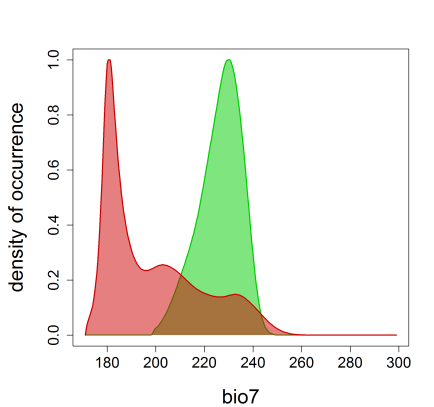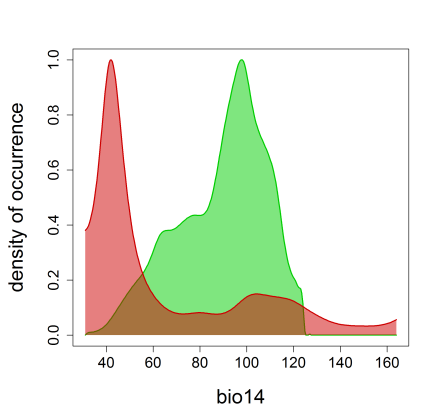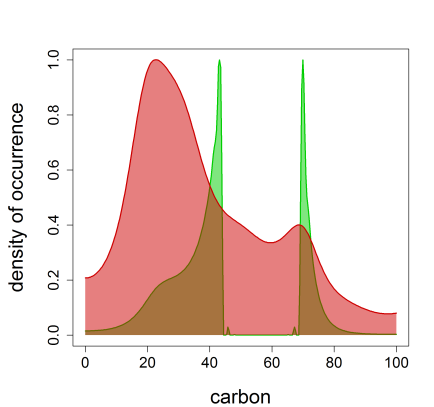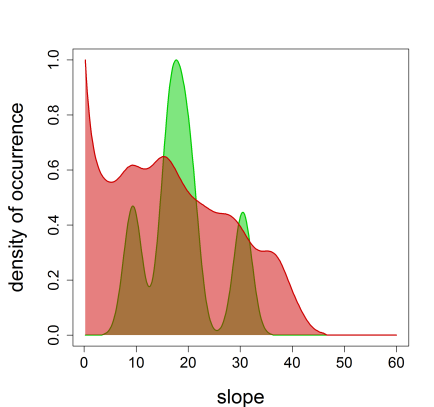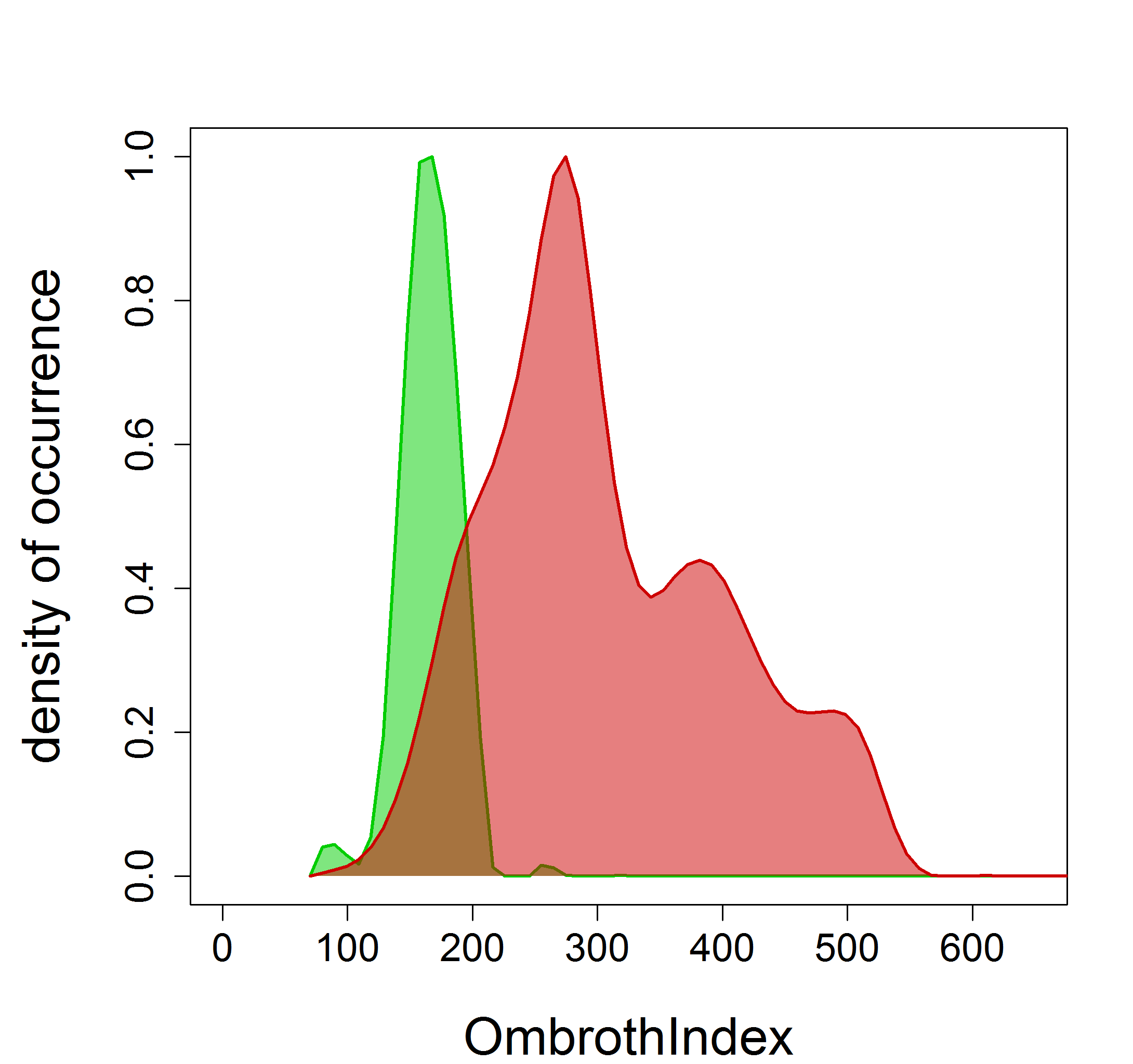 |
| --- | --- |
| **Fine-grained environmental variables** | 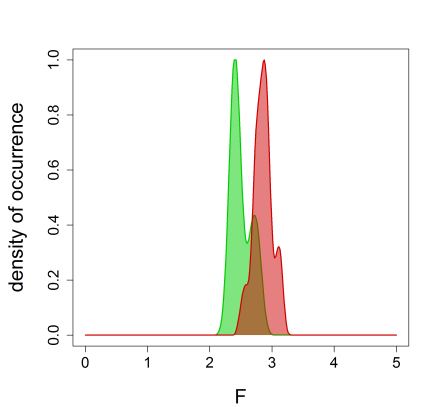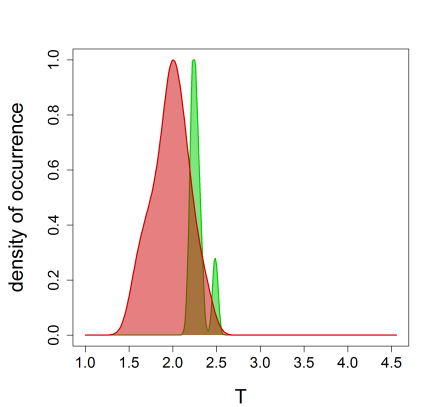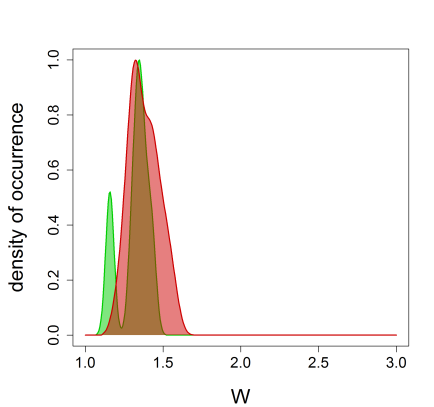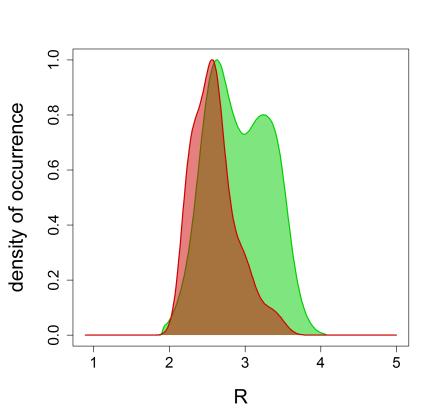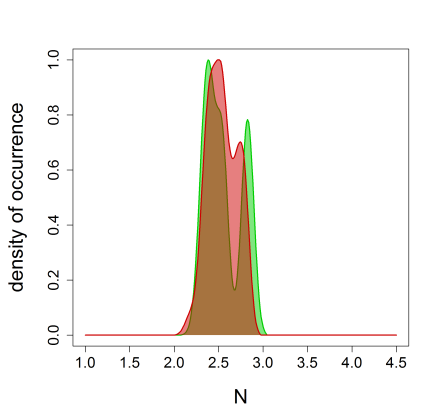 |

**Figure S4** Kernel density plots of the coarse-grained environmental and fine-grained environmental variables comparing comparing tetraploid *Ranunculus kuepferi* populations within the sympatric and outside the sympatric area. Densities of tetraploids within the sympatric and outside the sympatric area are shown in green and red respectively. Measurement units: temperature (°Cx10), precipitation (mm), slope (°), carbon (percentage area of calcareous substrates); for ombrothermic index see Appendix S1; for fine-grained environmental variables see descriptions in main text. The unusual carbon curve is the results of unavailable intermediary conditions in the sympatric area in our data.
